# Dose-Dependent Effects of TGF-β Inhibition on Osteoblast Differentiation and Wound Healing

**DOI:** 10.3390/cimb47050360

**Published:** 2025-05-14

**Authors:** Nihal Almuraikhi, Latifa Alkhamees, Sumaiya Tareen, Hessah Alshammari, Manikandan Muthurangan

**Affiliations:** 1 Stem Cell Unit, Department of Anatomy, College of Medicine, King Saud University, Riyadh 11461, Saudi Arabia; 442204106@student.ksu.edu.sa (L.A.); stareen@ksu.edu.sa (S.T.); mrangan@ksu.edu.sa (M.M.); 2 Cardiac Department, College of Medicine, King Saud University, Riyadh 11461, Saudi Arabia; healshammari@ksu.edu.sa

**Keywords:** TGF-β signaling, human bone marrow MSCs, osteoblast differentiation, wound healing

## Abstract

TGF-β is a multifunctional pathway that controls significant cellular and physiological processes and several pathological activities. TGF-β-induced signaling can be triggered upon binding to specific receptors to initiate the transcriptional activation of several genes and cellular processes. However, the detailed role of TGF-β signaling in osteoblast differentiation remains to be explicated. SB525334, a selective TGF-βRI inhibitor, was investigated for its effect on the osteoblastic differentiation of human bone marrow MSCs at different concentrations. Alkaline phosphatase (ALP) activity was used to assess osteoblast differentiation marker, while Alizarin red staining was used as a marker for mineralization. Expressions of osteoblast-specific genes were evaluated using real-time PCR. A migration assay was performed to assess the effect of TGF-β on wound healing. Moreover, immunofluorescent staining for SMAD2/3 and SMAD4 was employed to confirm the activation of the TGF-β pathway. The inhibition of TGF-β1 signaling using a high concentration of SB525334 (3 µM) significantly reduced ALP activity and mineralization and downregulated osteoblast-specific genes. However, the opposite effect was reported using a lower concentration (0.03 µM), where osteoblast-associated genes were significantly upregulated, and ALP activity and mineralization were higher. Significant scratch/wound healing was achieved at a lower concentration of SB525334, while a higher concentration of SB525334 resulted in lower healing. Moreover, a low concentration of SB525334 demonstrated nuclear translocation of SMAD 2/3 and 4. Our study confirms that the effect of TGF-β signaling in bone formation and wound healing is dose-dependent, and the use of TGF-β is recommended as a valuable therapeutic approach.

## 1. Introduction

Mesenchymal stem cells (MSCs) are adult stem cells residing in bone marrow and are able to differentiate into different types of stromal cells, including bone-forming osteoblasts [1]. The process of osteoblast formation is complicated and involves a crosslink between a number of signaling pathways, such as transforming growth factor β (TGF-β), Wingless/int1 (Wnt), bone morphogenetic proteins (BMPs), and tumor necrosis factor (TNF) [1,2,3,4,5,6,7]. However, their role in controlling MSCs’ differentiation into osteoblasts is not well established.

**Figure 1:**
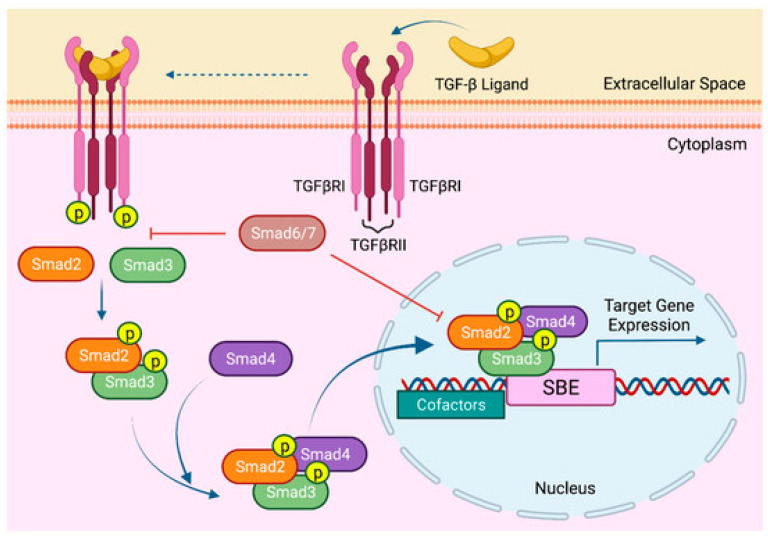
Canonical TGF-β signaling. Reproduced from Gungor et al., 2022 [Cancers, 14(4), 940], under Creative Commons Attribution (CC BY) license [19].

The TGF-β signaling pathway has a vital role in homeostasis, proliferation, differentiation, migration, and apoptosis [7,8,9]. It exists in all cell types in three different forms of receptor ligands with similar biological activities that can bind to three receptors (TGF-β receptor I (TGF-βRI), II, and III) also known as activin-like kinase (ALK) 5, ALK4, and Beta glycan, respectively [7,8,10]. In canonical TGF-β signaling, a TGF-β ligand binds to TGF-βRII, which phosphorylates TGF-βRI and stimulates its kinase activity (Figure 1). As a result, mothers against decapentaplegic homolog 2 and 3 (SMAD2 and 3) become phosphorylated, which then trigger the translocation of SMAD4 to the nucleus, where it can control the transcription of TGF-β target genes [7,8,10,11,12]. Previous studies confirmed the integral role of TGF-βRI in bone remodeling, regulating MSC differentiation into osteoblast differentiation and matrix formation [7,13,14,15]. Moreover, TGF-β cytokines is abundantly deposited in the bone matrix of mammals [15], and upon activation, it inhibits osteoblast differentiation [16,17] and stimulates osteoblast proliferation at bone remodeling sites [18].

Small molecule inhibitors of certain intracellular signaling pathways are widely engaged in investigating the molecular matrix regulating the osteogenic differentiation progression [1,2,3,4,5,6,7]. Herein, we identified SB525334, a TGF-βRI inhibitor, as a potent regulator of in vitro osteoblastic differentiation of human bone marrow MSCs, which makes it a potential therapeutic tool against bone-related disorders.

## 2. Materials and Methods

### 2.1. Cell Culture

Throughout this study, the human mesenchymal stem cell (hMSC)-TERT line was used as a model for hMSCs. These cells are human bone marrow MSCs generated by the overexpression of the human telomerase reverse transcriptase (hTERT) gene. These cells have been approved to conserve the unique features of primary hMSCs, including unlimited proliferation, potency, and gene expression [20,21]. To culture the cells, Dulbecco’s modified eagle medium (DMEM) was used as the basal medium, supplemented with 4500 mg/L D-glucose, 4 mM L-glutamine, and 110 mg/L of 10% sodium pyruvate, 10% fetal bovine serum (FBS), 1% nonessential amino acids, and 1% penicillin-streptomycin, as described by Simonsen et al. [20]. All supplements were obtained from Thermo Fisher Scientific Life Sciences (Waltham, MA, USA) (https://www.thermofisher.com, accessed on 2 December 2024). Cells were incubated at 37 °C with 95% humidity and 5% CO_2_ until they reached confluency.

### 2.2. Osteoblast Differentiation

Cells were cultured to attain 70-80% confluency; for osteoblast differentiation, media substituted with 10% FBS, 1% penicillin-streptomycin, 10 nM calcitriol, 10 nM dexamethasone, and 10 mM b- glycerophosphate, all from Sigma-Aldrich (St. Louis, MO, USA), and 50 mg/mL L-ascorbic acid (Wako Chemicals; https://www.wako-chemicals.de/, accessed on 2 December 2024) were used as described by AlMuraikhi et al. [2]. The small-molecule TGF-β inhibitor (SB525334) was purchased from Selleckchem Inc. (Houston, TX, USA) (Cat. No L2100) (https://www.selleckchem.com, accessed on 2 December 2024), which was then added to the osteoblast differentiation medium at a concentration of 3 µM with constant exposure throughout the differentiation periods. For control cells, dimethyl sulfoxide (DMSO) was used as a vehicle in the osteoblast differentiation medium.

### 2.3. Cell Viability Assay and Dose–Response Proliferation Curve

AlamarBlue test was performed according to the manufacturer’s instructions (Thermo Fisher Scientific), as described by AlMuraikhi et al. [2]. For the dose–response proliferation curve, cells were cultured in 96-well plates with 300 μL of medium and supplemented with 0.03, 0.3, and 3 μM of small molecule inhibitor (SB525334), and DMSO was used for the control cells. Then, 30 μL (approx... 10%) of alamarBlue was added to cells, and they were kept at 37 °C for 2 h. Plates were read at Wx 530 nm/Em 590 nm using a BioTek Synergy II microplate reader (BioTek Inc., Winooski, VT, USA) for Days 1, 2, and 3. For cell viability assay, similar culture conditions were applied on the cells, but 30 μL/well of AlamarBlue was added on Day 10, and then cells were kept at 37 °C for 1 h. Subsequently, readings were acquired in the same way as the dose–response proliferation curve.

### 2.4. Measurement of Apoptosis

Apoptosis was determined by acridine orange/ethidium bromide (AO/EtBr) fluorescence staining, where cells were exposed to 0.03 µM and 3 µM of SB525334 and DMSO control [1,22]. On Day 3, cells that were exposed 0.03 µM and 3 µM of SB525334 and DMSO control were stained with dual fluorescent staining solution containing 100 µg/mL AO and 100 µg/mL EtBr (AO/EB, Sigma) for 1 min, and then images were taken with a Nikon Eclipse Ti fluorescence microscope (Nikon, Tokyo, Japan).

### 2.5. Quantification of Alkaline Phosphatase Activity

Alkaline phosphatase (ALP) enzyme activity was measured using an ALP activity colorimetric assay kit (BioVision Inc., Milpitas, CA, USA) with some modifications, as described by AlMuraikhi et al. [2]. Cells were cultured in osteoblast differentiation medium in a 96-well plate for 10 days. Then, cells were washed with PBS and fixed with 3.7% formaldehyde in 90% ethanol for 30 s at room temperature, and the fixative was discarded and incubated with 50 µL/well of a p-nitrophenyl phosphate solution for 60 min. At the end of the incubation period, optical density (OD) was measured at 405 nm using a SpectraMax/M5 fluorescence spectrophotometer plate reader.

### 2.6. Alkaline Phosphatase Staining

ALP staining was performed on cells that were cultured with osteoblast induction medium for 10 days, as previously described by AlMuraikhi et al. [2]. Cells were fixed using 10 mM acetone/citrate buffer (pH 4.2) for 5 min at room temperature and stained with Naphthol/Fast Red stain, Sigma (0.2 mg/mL Naphthol AS-TR phosphate substrate) (0.417 mg/mL of Fast Red) for 1 h at room temperature. Then, images were taken under a microscope. All conditions were achieved in biological duplicates, images were taken from three fields/well, and a representative image is shown.

### 2.7. Alizarin Red S Staining for Mineralized Matrix Formation

Alizarin red staining was performed on cells cultured in osteoblast differentiation medium for 21 days, as previously described by AlMuraikhi et al. [2]. Cells were fixed using 4% paraformaldehyde for 10 min at room temperature and stained with the Alizarin Red S (2%) Staining Kit (Cat. No. 0223, ScienceCell, Research Laboratories) for 30 min at room temperature. Then, images were taken under a microscope. All conditions were achieved in biological duplicates, images were taken from three fields/well, and a representative image is shown.

### 2.8. RNA Extraction and cDNA Synthesis

RNA extraction from cells cultured in osteoblast differentiation medium was performed on Day 10 for two biological replicates using PureLink Kit (Cat. No.: 12183018A, Ambion by Life Technologies, Carlsbad, CA, USA) based on the manufacturer’s instructions, as previously described by AlMuraikhi et al. [2]. Cells were lysed with lysis buffer, and the protein in the lysate was precipitated by ethanol, transferred to a spin column, and washed with washing buffers. Finally, RNA was eluted in nuclease-free water, and the concentration was measured using NanoDrop 2000 (ThermoFisher Scientific Life Sciences). Afterward, cDNA synthesis was performed with 500 ng of RNA using High-Capacity cDNA Transcription Kit (Cat. No 4368814, ThermoFisher Scientific Life Sciences) based on the manufacturer’s instructions.

### 2.9. Quantitative Real-Time Polymerase Chain Reaction (RT-PCR)

The Biosystems ViiA™ 7 real-time PCR System (ThermoFisher Scientific Life Sciences) was used to perform quantitative RT-PCR using fast SYBR Green (Cat. No 4385612). All reactions were performed in technical triplicate from biological duplicates. All primers used in this study are listed in Table 1. Relative gene expression was calculated using the 2Δ CT value, and RT-PCR was performed as previously described [23].

### 2.10. Migration/Scratch Assay 

The migration test was performed on SB525334-treated hMSCs only since TGFβ has been shown to increase wound healing in vitro [24]. Therefore, hMSCs were cultivated in a 12-well plate and treated with 0.03 µM and 3 µM of SB525334 and DMSO, the latter of which was used as a control. Cells (0.1 × 106/well) were seeded in 12-well plates for 24 h to achieve desired confluency. A sterile ruler was used to reference the center, and an artificial scratch wound was produced in each well using a sterile 200 µL pipette tip; after that, the wells were washed with PBS to remove debris, and wound closure was assessed at 12, 24, and 36 h using a light microscope (Carl Zeiss Canada, North York, ON, Canada). Images were obtained using Zen software (Carl Zeiss Canada, North York, ON, Canada). Then, wound healing area percentages were calculated using ImageJ software (Version 1.53) (U.S. National Institutes of Health, Bethesda, MD, USA) at three time points as previously described: 0 to 12 h, 12 to 24 h, and 24 to 36 h. Data are representative of 3 replicas for each experimental condition, where 3 images were captured for each condition [25].

### 2.11. Immunocytochemistry

SB525334-treated human MSCs were grown on sterile glass coverslips. The cells were washed twice with PBS and fixed using 4% paraformaldehyde for 15 min at room temperature. The cells were then permeabilized with 0.1% Triton X-100 in PBS for 10 min and blocked using blocking serum 4.5% (bovine serum albumin (BSA) in PBS) for 30 min. Next, the cells were incubated with primary antibodies (SMAD2/3 and SMAD4—Rabbit IgG) overnight at 4 °C. After incubation, the cells were washed three times with PBS for 5 min each. Secondary primers (Goat Anti-Rabbit IgG from Abcam) were added and incubated for 1 h in the dark. The cells were washed thrice with PBS and counterstained with DAPI for 5 min. Images were taken using a Nikon Eclipse Ti fluorescence microscope.

### 2.12. Statistical Analysis

Microsoft Excel and GraphPad Prism 6.0 software were used for statistical analysis and graphing, respectively. Results are shown as the mean ± SEM of at least two independent experiments. All statistical significance was determined either using an ANOVA followed by Dunnett’s post hoc test or using a multiple *t*-test with multiple comparisons using the Holm–Sidak method, and *p* < 0.05 was set to indicate statistical significance.

## 3. Results

### 3.1. SB525334 Has no Suppressive Effect on Human MSC Proliferation

**Figure 2:**
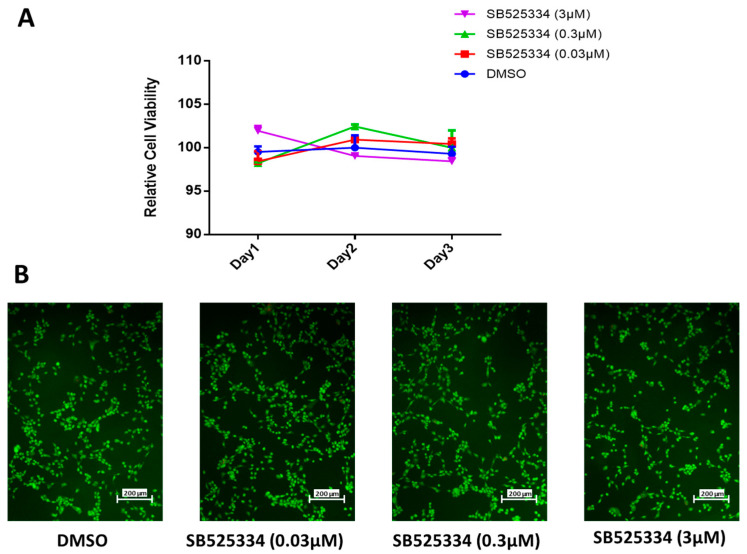
Effects of SB525334 treatment on viability of human MSCs. (**A**) Dose–response proliferation curve of human MSCs to different doses of SB525334 treatment, as indicated in graph, versus DMSO-treated control cells as measured by cell viability over 3 days. (**B**) Representative fluorescence images of human MSCs treated with 0.03 µM, 0.3 µM, or 3.0 µM of SB525334 versus DMSO-treated control cells 3 days after exposure. Photomicrographs with magnification of ×10. Cells were stained with AO/EtBr to detect apoptotic cells (cells with green condensed chromatin) and necrotic cells (red).

In preceding studies, we described different effects of small molecule inhibitors on the osteoblast differentiation of human MSCs based on the screening of a small molecule inhibitor library referring to ALP activity quantification as a read-out (2). Among these, SB525334, a TGF-β type I receptor inhibitor, exhibited an opposite effect at different concentrations, which led to a further investigation. First, we evaluated the effect of the in vitro treatment of SB525334 at a logarithmic scale at concentrations of 0.03, 0.3, and 3 µM for 1, 2, and 3 days on human MSC proliferation (Figure 2A). all concentrations confirmed no significant effects of SB525334 on human MSC proliferation. Furthermore, an apoptosis test was performed after 3 days of SB525334 treatment at concentrations of 0.03, 0.3, and 3 µM. However, there were no significant variations detected in apoptotic and necrotic cells compared to the DMSO-vehicle-treated control cells (Figure 2B).

### 3.2. SB525334 Exhibited an Opposite Effect on the Osteoblast Differentiation of Human MSCs

**Figure 3:**
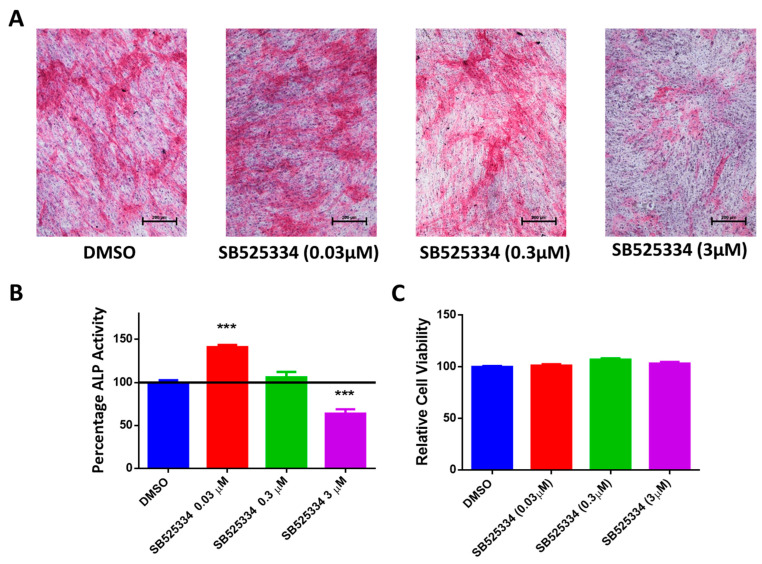
Effects of SB525334 treatment on ALP activity and staining of human MSCs. (**A**) Representative alkaline phosphatase (ALP) staining of SB525334-treated human MSCs at 0.03 µM, 0.3 µM, or 3.0 µM versus DMSO-treated control cells on Day 10 post-osteoblast differentiation. Photomicrographs with magnification of ×10. (**B**) Quantification of ALP activity in SB525334-treated human MSCs at 0.03 µM, 0.3 µM, or 3.0 µM versus DMSO-treated control cells on Day 10 post-osteoblast differentiation. Data are presented as mean percentage of ALP activity ± SEM (n = 20); *** *p* ≤ 0.0005. (**C**) Assay for cell viability using Alamar Blue assay in SB525334-treated human MSCs at 0.03 µM, 0.3 µM, or 3.0 µM versus DMSO-treated control cells on Day 10 post-osteoblast differentiation. Data are presented as mean ± SEM (n = 20).

SB525334-treated human MSCs at a 0.03 µM concentration exhibited a significant increase in ALP formation, as demonstrated by an increase in cytochemical staining intensity (Figure 3A), which was confirmed by an elevation in the ALP activity measurement on Day 10 of osteoblast differentiation (Figure 3B). On the other hand, SB525334-treated human MSCs at a 3 µM concentration exhibited a significant decrease in ALP formation, as demonstrated by an increase in cytochemical staining intensity (Figure 3A), which was confirmed by a reduction in the ALP activity measurement on Day 10 of osteoblast differentiation (Figure 3B). SB525334-treated human MSCs at a 0.3 µM concentration exhibited insignificant ALP formation (Figure 3A), which was confirmed by the ALP activity measurement on Day 10 of osteoblast differentiation (Figure 3B), and thus, this concentration was excluded from the study. Moreover, at all concentrations, SB525334 did not show a significant effect on human MSC viability on Day 10 of osteoblast differentiation (Figure 3C).

**Figure 4:**
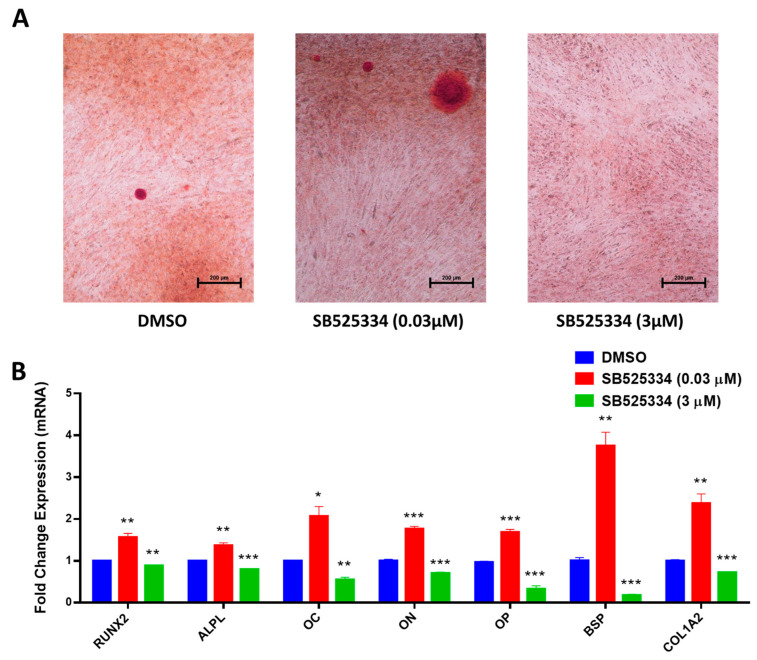
Effects of SB525334 treatment on mineralization and gene expression of human MSCs. (**A**) Cytochemical staining for mineralized matrix formation using Alizarin red staining on Day 21 post-osteoblast differentiation on human MSCs treated with 0.03 µM and 3.0 µM of SB525334. Photomicrographs with magnification of ×10. (**B**) Quantitative RT-PCR analysis for gene expression of RUNX2, ALPL, OC, ON, OP, BSP, and COL1A2 in human MSCs treated with 0.03 µM and 3.0 µM SB525334 on Day 10 post-osteoblast differentiation. Gene expression was normalized to β-actin. Data are presented as mean fold change ± SEM (n = 6) from two independent experiments; * *p* < 0.05; ** *p* < 0.005; *** *p* ≤ 0.0005.

SB525334-treated human MSCs at a 0.03 µM concentration revealed a significant elevation in matrix production, as assessed by mineralized nodule formation using Alizarin red staining (Figure 4A), together with a significant upregulation in the expression of several osteoblast gene markers: runt-related transcription factor 2 (RUNX2), ALPL, Osteocalcin (OC), Osteonectin (ON), Osteopontin (OP), bone sialoprotein (BSP), and Collagen Type I Alpha 2 (COL1A2) (Figure 4B). In contrast, SB525334-treated human MSCs at a 3 µM concentration revealed no mineralized matrix production, as assessed by Alizarin red staining (Figure 4A), together with a significant downregulation in the expression of the abovementioned osteoblast gene markers (Figure 4B).

### 3.3. Effect of SB525334 on TGF-β Signaling Pathway

**Figure 5:**
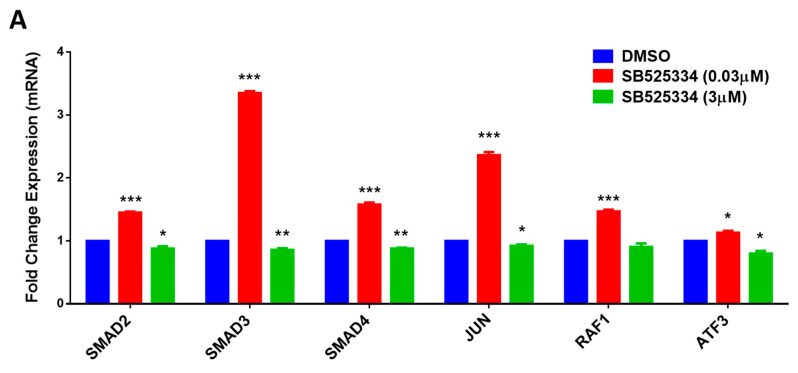

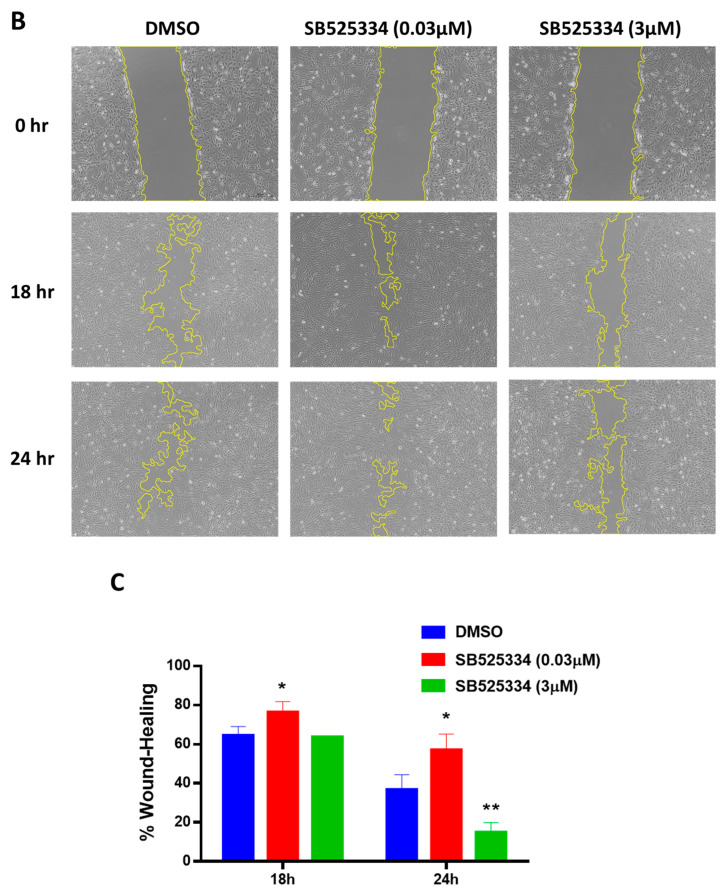

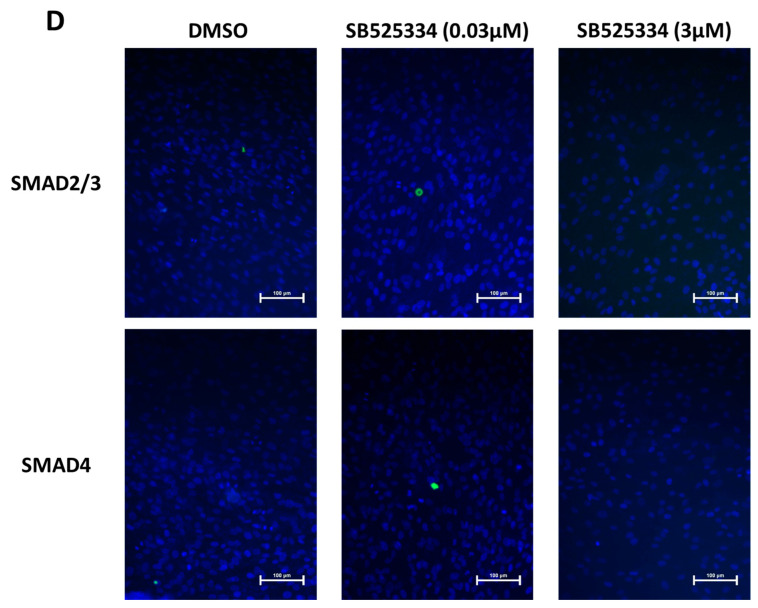
Effect of SB525334 on TGF-β signaling pathway. (**A**) Quantitative RT-PCR analysis for gene expression of TGF-β signaling-associated genes, including SMAD2, SMAD3, SMAD4, JUN, RAF1, and ATF3, in human MSCs treated with 0.03 µM and 3.0 µM of SB525334 on Day 10 post osteoblast differentiation. Gene expression was normalized to β-actin. Data are presented as mean fold change ± SEM (n = 6) from two independent experiments; * *p* < 0.05; ** *p* < 0.005; *** *p* ≤ 0.0005. (**B**) Analysis of scratch assay in human MSCs treated with 0.03 µM and 3.0 µM of SB525334 18 and 24 h post scratch. Healing was measured using ImageJ software (Version 1.53) with MRI Wound Healing Tool and analyzed using Prism GraphPad. Images were taken at 10× magnification using AxioCam MRc5 (ZEISS). *p*-value: * *p* < 0.0005. (**C**) Percentages of wound closure in human MSCs treated with 0.03 µM and 3.0 µM of SB525334 18 and 24 h post scratch; * *p* < 0.05; ** *p* < 0.005. (**D**) Immunocytochemistry staining/imaging in human MSCs treated with 0.03 µM and 3.0 µM of SB525334 at Day 10 post osteoblast differentiation for SMAD2/3 and SMAD4. Images were taken using Nikon Eclipse Ti fluorescence microscope at 10× magnification.

To confirm that SB525334 targets the TGF-β signaling pathway, human MSCs were treated with SB525334 at both concentrations (0.03 and 3 μM), and after 48 h, the expressions of TGF-β signaling-associated genes, including SMAD2, SMAD3, SMAD4, JUN, RAF1, and ATF3, were assessed using qRT-PCR. The data shown in Figure 5A demonstrate a significant increase in TGF-β-associated genes at a 0.03 μM concentration of SB525334, while all genes were significantly decreased at a 3 μM concentration of SB525334. These data indicate that SB525334 affects the osteoblast differentiation of human MSCs through the TGF-β signaling pathway.

Moreover, in vitro wound healing was found to be improved by the TGF-β signaling pathway in a dose-dependent manner [26]. In this study, a scratch-wound assay was performed to further confirm the effect on the TGF-β signaling pathway by the subsequent cellular mechanisms in response to treating human MSCs with SB525334. As shown in Figure 5B,C, the results are consistent with previous observations, with early scratch/wound-healing achieved at a 0.03 µM concentration of SB525334 compared to the DMSO-treated control cells, and the results were significantly higher at the 18 h and 24 h time points. As for human MSCs treated with a 3 µM concentration of SB525334, wound healing was delayed at both time points, and it was significantly lower at 24 h compared to the DMSO-treated control cells.

Furthermore, SMAD2/3 and SMAD4 are key players in TGF-β downstream signaling [27]. Qualitative immunofluorescent staining for SMAD2/3 and SMAD4 was carried out to confirm the involvement of the TGF-β pathway. Immunohistofluorescence microscopy imaging visualized higher immunofluorescence intensity for SMAD2/3 and SMAD4 proteins in human MSCs treated with 0.03 µM of SB525334 compared to human MSCs treated with 3 µM of SB525334 and DMSO-treated control cells (Figure 5D).

## 4. Discussion

TGF-β is a multifunctional pathway that controls key cellular processes, such as proliferation, differentiation, and apoptosis, and physiological processes, such as wound healing, in addition to different pathological progression such as fibrosis, cancer, and metastasis [15,28]. TGF-β-induced signaling is triggered when the TGF-β ligand binds to TGF-βRII, which then activates TGF-βRI to phosphorylate Smad 2 and 3, releasing them from the receptor complex. Smad 2 and 3 then bind to smad4 to translocate to the nucleus to initiate the transcriptional activation of several genes and cellular processes [29,30,31].

TGF-β has been shown to act in a context-dependent manner in many conditions; for example, in tumor progression, it acts as a tumor promoter, enhancing cell expansion and migration to metastasize, as it undergoes mutation and epigenetic modulation to become deactivated with no usual suppressive effect on cancer cells [32,33,34,35,36,37,38]. On the other hand, TGF-β suppresses the immune system in recognizing tumor cells [28]. Thus, this unique nature of TGF-β makes it a good candidate for a variety of pre-clinical and clinical studies to assess tumor-suppressing or tumor-promoting responses to TGF-β signaling-associated therapies in different types of cancers [28]. TGF-β has also shown an involvement in bone remodeling and matrix formation [7]. Thus, the inhibition of TGF-β has also been widely proposed as a therapeutic tool for many bone-related pathological models for a better understanding of its role and regulation in osteogenesis. In this study, using small molecule library screening, we presented an opposite effect of a small molecule, SB525334, which is a TGF-βRI inhibitor, on the in vitro osteoblast differentiation of human MSCs.

TGF-β plays a pivotal role in bone remodeling, and its dysregulation is implicated in various bone-related disorders. In osteolytic bone metastases, TGF-β released during bone resorption enhances tumor growth and furthers bone degradation; thus, inhibiting TGF-β can disrupt this cycle, reducing tumor burden and bone destruction [39]. In osteogenesis imperfecta (OI), characterized by brittle bones, excessive TGF-β signaling contributes to bone fragility; studies indicate that TGF-β inhibition can increase bone volume and strength in OI models [40]. Similarly, in osteoarthritis (OA), elevated TGF-β levels in subchondral bone lead to pathological changes; targeted inhibition has been shown to attenuate OA progression by normalizing bone remodeling [41]. Furthermore, in multiple myeloma, TGF-β supports disease progression and associated osteolytic bone disease; blocking TGF-β activation has been shown to reduce tumor burden and bone destruction [42,43].

SB525334 is a selective inhibitor of TGF-βRI (ALK5) with a lesser inhibitory effect on ALK4, but not other kinases, including ALK3 and ALK6 [11]. SB525334 has shown an anti-fibrotic effects in different pathological models associated with the TGF-β signaling pathway, such as nephropathy [44,45], pulmonary fibrosis [46], and hepatic injury [47], as well as ovarian cancer [48], pancreatic cancer [49], and hepatocellular carcinoma [50].

SB525334 is known to block TGF-β-induced Smad2/3 phosphorylation and subsequent signaling pathways [51]. The dual effects observed with SB525334 treatment can be attributed to its concentration-dependent modulation of TGF-β signaling. At low concentrations, the partial inhibition of TGF-βRI may reduce the activation of inhibitory Smads, thereby relieving their suppression on osteogenic transcription factors such as Runx2. This partial blockage allows for the elevation of osteoblast differentiation and matrix formation [52]. However, at high concentrations, the complete inhibition of TGF-βRI occurs, which leads to a significant reduction in Smad2/3 phosphorylation, effectively downregulating TGF-β signaling. This comprehensive blockade hampers osteoblast differentiation and diminishes matrix mineralization [52].

These findings underscore the importance of carefully titrating SB525334 concentrations to harness its therapeutic potential in bone-related disorders. By optimizing the dosage, it may be possible to effectively promote bone formation while minimizing adverse effects associated with the excessive inhibition of TGF-β signaling.

Previous studies have described that the inhibition of TGF-β1 signaling with different TGF-β1 inhibitors, SB431542 and SB505124, significantly reduced mineralization deposits and downregulated osteoblast-associated genes, which partially agrees with the current results obtained with the higher concentration (3 µM). However, we reported an opposite effect at a lower concentration (0.03 µM), where osteoblast-associated genes were significantly upregulated, and the matrix formation was higher.

Moreover, our data are in agreement with previous studies that suggested the importance of TGF-β1 in wound healing, as significant scratch/wound healing was achieved with a lower concentration of SB525334 [24,26]. On the other hand, the higher concentration of SB525334 demonstrated significantly lower healing compared to the control, confirming that the effect of the TGF-β signaling pathway on wound healing occurs in a dose-dependent manner, where a low concentration of TGF-β1 reduces SMAD2/3 nuclear localization and stimulates p38MAPK, a key to cellular migration, thus promoting cell migration and wound closure [26]. Moreover, the low concentration of SB525334 demonstrated nuclear translocation of SMAD 2/3 and 4, suggesting that the low concentration was insufficient to inhibit TGF-β signaling.

Future research should focus on developing bone-targeted TGF-β inhibitors to minimize systemic side effects, possibly through advanced drug delivery systems or bone-seeking compounds. Additionally, combination therapies that pair TGF-β inhibitors with existing treatments, such as anti-resorptives or anabolic agents, warrant further investigation to assess potential synergistic effects. Long-term studies are essential to evaluate the safety and efficacy of TGF-β inhibition, particularly concerning its impact on normal bone remodeling and fracture healing. Furthermore, identifying biomarkers to predict patients’ response to TGF-β-targeted therapies could facilitate personalized treatment approaches. Finally, elucidating the specific roles of different TGF-β isoforms in bone pathology may lead to more selective and effective therapeutic strategies [53].

In conclusion, our study confirms the involvement of TGF-β signaling in bone formation and that the inhibition of TGF-β could be a valuable approach. Dosing may need to be optimized in order to match the therapeutic purpose.

## Figures and Tables

**Table 1:** A list of SYBR Green primers used in the present study.

Gene Name	Forward Primer	Reverse Primer
ACTB	5′AGCCATGTACGTTGCTA	5′AGTCCGCCTAGAAGCA
ALPL	5′GGAACTCCTGACCCTTGACC3′	5′TCCTGTTCAGCTCGTACTGC3′
OC	GGCAGCGAGGTAGTGAAGAG	CTCACACACCTCCCTCCTG
ON	5′GAGGAAACCGAAGAGGAGG3′	5′GGGGTGTTGTTCTCATCCAG3′
RUNX2	5′GTAGATGGACCTCGGGAACC3′	5′GAGGCGGTCAGAGAACAAAC3′
OP	GGTGATGTCCTCGTCTGTA	CCAAGTAAGTCCAACGAAAG
BSP	GCAGTAGTGACTCATCCGAAGAA	GCCTCAGAGTCTTCATCTTCATTC
COL1A2	GGTCTTCCAGGCCTCTCC	ACCCTTGGCACCAGTAAGG
SMAD2	TGCTCTGAAATTTGGGGACTGA	ACGACCATCAAGAGACCTGG
SMAD3	TAATTTATTGCCGCCGCTCG	GGCCATCCAGGGACTCAAAC
SMAD4	ATTTGCCTCACCACCAAAAC	AGCAGGATGATTGGAAATGG
JUN	CAGCCAGGTCGGCAGTATAG	GGGACTCTGCCACTTGTCTC
RAF1	CCTGGCTCCCTCAGGTTTAAG	TGATCGTCTTCCAAGCTCCC
ATF3	GTGAGTCCTCGGTGCTCG	GCATCATTTTGCTCCAGGCT

## Data Availability

No new data were created or analyzed in this study. Data sharing is not applicable to this article.

## Abbreviations

The following abbreviations are used in this manuscript:

MSCs	Mesenchymal stem cells
TGF-β	Transforming growth factor β
TGF-βR	TGF-β receptor
ALK	Activin-like kinase
SMAD	Mothers against decapentaplegic homolog
ALPL	Alkaline phosphatase
DMEM	Dulbecco’s modified eagle medium
DMSO	Dimethyl sulfoxide
hTERT	Human telomerase reverse transcriptase
PBS	Phosphate-buffered saline
qRT-PCR	Quantitative reverse transcriptase polymerase chain reaction
BMP	Bone morphogenetic proteins
Wnt	Wingless/int1
TNF	Tumor necrosis factor
Runx2	Runt-related transcription factor 2
OC	Osteocalcin
ON	Osteonectin
OP	Osteopontin
BSP	Bone sialoprotein
COL1A2	Collagen type I alpha 2
JUN	Jun proto-oncogene
RAF1	Raf-1 proto-oncogene
ATF3	Activating transcription factor 3
